# Improving the clinical potential of ultra-high field fMRI using a model-free analysis method based on response consistency

**DOI:** 10.1007/s10334-016-0533-8

**Published:** 2016-03-10

**Authors:** Pedro Lima Cardoso, Florian Ph. S. Fischmeister, Barbara Dymerska, Alexander Geißler, Moritz Wurnig, Siegfried Trattnig, Roland Beisteiner, Simon Daniel Robinson

**Affiliations:** Department of Biomedical Imaging and Image-guided Therapy, High Field Magnetic Resonance Centre, Medical University of Vienna, Lazarettgasse 14/BT32, 1090 Vienna, Austria; Study Group Clinical fMRI, Department of Neurology, Medical University of Vienna, Währinger Gürtel 18-20, 1090 Vienna, Austria

**Keywords:** fMRI analysis, Modified BOLD response, Ultra-high field, Presurgical planning, UNBIASED

## Abstract

**Objective:**

To develop an analysis method that is sensitive to non-model-conform responses often encountered in ultra-high field presurgical planning fMRI. Using the consistency of time courses over a number of experiment repetitions, it should exclude low quality runs and generate activation maps that reflect the reliability of responses.

**Materials and methods:**

7 T fMRI data were acquired from six healthy volunteers: three performing purely motor tasks and three a visuomotor task. These were analysed with the proposed approach (UNBIASED) and the GLM.

**Results:**

UNBIASED results were generally less affected by false positive results than the GLM. Runs that were identified as being of low quality were confirmed to contain little or no activation. In two cases, regions were identified as activated in UNBIASED but not GLM results. Signal changes in these areas were time-locked to the task, but were delayed or transient.

**Conclusion:**

UNBIASED is shown to be a reliable means of identifying consistent task-related signal changes regardless of response timing. In presurgical planning, UNBIASED could be used to rapidly generate reliable maps of the consistency with which eloquent brain regions are activated without recourse to task timing and despite modified hemodynamics.

**Electronic supplementary material:**

The online version of this article (doi:10.1007/s10334-016-0533-8) contains supplementary material, which is available to authorized users.

## Introduction

The General Linear Model (GLM) [1] is a simple but powerful method for identifying task-related activation in functional MRI (fMRI) and has become the dominant approach used in cognitive and clinical neuroscience. A number of exceptions to the assumptions underpinning the GLM have been documented, however. The Hemodynamic Response Function (HRF) is known to vary between brain regions and participants [2] and is altered close to pathologies [3–5]. Blood oxygenation level-dependent (BOLD) signal changes may be negative [6, 7] or transient [8], and the response shape varies throughout the brain [9]. These potential confounds and the difficulty in accurately recording task processing (i.e. if and when the task was executed) in a number of contexts provide the motivation for the development of analysis methods which do not share the GLM’s assumptions about the timing, shape or course of BOLD signal changes.

Model-based fMRI methods are based on assumptions about the timing of neural events, the shape of the HRF, and linearity in the response. We begin by summarizing the known difficulties raised by these assumptions before briefly reviewing alternative approaches.

Task processing may occur at unanticipated times or with unexpected duration. There may also be no suitable MR-compatible equipment capable of recording the responses of interest, particularly at very high static magnetic field. In clinical studies, patients frequently have problems responding promptly to tasks or may not react consistently over trials. In practice, performance in a clinical setting is usually monitored visually by subjective or semi-quantitative methods, which introduces error into the estimation of the stimulus function [10].

Variability in the shape of the hemodynamic response across subjects and regions can reduce BOLD sensitivity even in healthy populations [2]. Developmental differences [11, 12] and consumption of vasoactive substances such as caffeine can also change the temporal dynamics of the BOLD response [13]. In a clinical context, neurovascular uncoupling has been reported in both low grade [4] and high grade brain tumours, leading to activation going undetected [5]. Furthermore, the time-to-peak of the HRF may be modified in regions of pathology [14] and the concentration of deoxyhemoglobin may even increase in response to activation, contrary to the typical behaviour [3]. Cerebrovascular diseases and arteriovenous malformations (AVMs) have also been reported to lead to a reduction of the BOLD response in the motor cortex ipsilateral to the stenosis [15] and to reduced regional cerebral blood flow (rCBF) and perfusion [16], respectively, modifying the HRF. This leads to variability in motor cortex activation patterns among brain tumour patients [17] and a potential inability to detect viable neuronal tissue [18].

An expanding body of literature has documented sustained negative [6, 7], phasic [19], and transient [8] cerebral blood flow (CBF) and BOLD responses, and there has been a resurgence of interest in the variability and reproducibility of BOLD responses to relatively simple tasks over a large number of repetitions [9]. These document responses that are reproducible, but do not conform to a standard model. Such discrepancies may be expected to be different between healthy and clinical populations [20].

Many of these potential confounds are encountered in fMRI for presurgical planning. Patients are less likely to be able to adhere to prescribed timing, and the presence of pathologies modifies the HRF. Model-free analysis offers potential solutions. Independent Component Analysis (ICA), for instance, does not require assumptions about task timing, the shape of the HRF, or linearity in the response [21]. Despite substantial advantages over the GLM in the context of presurgical planning at ultra-high field (UHF) [22], ICA has found little application to date, though, due to the need to assess and interpret the large number of components (ICs) generated.

Levin and Uftring suggested an alternative model-free method called Biasless Identification of Activated Sites by Linear Evaluation of Signal Similarity (BIASLESS) [23], based only on the assumption that in the same individual the signal time courses in activated voxels show similar fluctuations in repetitions of the same experiment. This concept was extended to an inter-subject level in an attempt to investigate the similarity of cortical responses in different individuals during natural vision processing [24]. However, BIASLESS has found relatively little application in modern fMRI experiments, as these tend to use event-related designs with randomized and jittered timing and a range of stimuli. Preoperative fMRI, on the other hand, is often carried out as repeated executions of paradigms (runs) with identical tasks and timing [25]. Increasing the fMRI duration (e.g. via the number of runs) increases the statistical power of the analysis. The separation of the total fMRI period into runs allows patients short rest periods and enables the reliability of activation to be assessed (e.g. [26]). These features make this field open to analysis with an approach based on the reproducibility of responses. It is, therefore, expected to benefit from a method robust to consistent delays, modified HRF and reproducible aberrant BOLD response. Interest in this direction has been reawakened. Building upon the clinically established “risk map” approach [26], Stevens et al. showed that the reliability of fMRI presurgical mapping may be improved via optimisation of preprocessing pipelines using a within-session test–retest acquisition [27].

Since the acquisition of fMRI data at UHF strength provides increased time-series signal-to-noise ratio (SNR), BOLD sensitivity [28–30], and specificity to BOLD signal changes in the microvasculature [31], clinical populations might particularly benefit from the possibility to reduce the measurement time and improve the reliability of activation detection.

The aim of this study was to extend the BIASLESS approach of Levin and Uftring [23], which used cross-correlation between just two runs, to a method capable of utilizing information from N runs (*N* ≥ 2), to introduce a fully automated means of identifying and excluding low quality runs and to generate results which are an index of the reliability of the response. This extended method, which we call UNBIASED, is tested for reproducibility, sensitivity to shifts in response timing, and the ability to identify poor runs in a range of motor and visual tasks in healthy subjects at 7 T. Differences in results originating from UNBIASED and GLM analyses are investigated. Cortical regions with responses that were consistent, but not model-conform were identified with UNBIASED, but not the GLM.

## Materials and methods

The steps in UNBIASED are explained in “UNBIASED analysis”. Experiments with healthy subjects, described in “Task description”, assess the reproducibility of results over a range of motor tasks, robustness to consistent timing errors, and the ability of the approach to exclude “bad” runs. UNBIASED was also applied to functional localization of the primary motor and visual cortex in these volunteers and regions of modified response shape were investigated. Results were compared with those obtained with a GLM.

All the analysis was implemented in MATLAB (Mathworks Inc, Natick, MA, USA), unless otherwise stated. An in-house implementation of the GLM (ihGLM) was used to allow direct comparison of quality of fit between GLM and UNBIASED for data preprocessed with the same scheme. Otherwise, GLM analyses were performed with SPM8 (http://www.fil.ion.ucl.ac.uk/spm) (see “GLM analysis”).

### UNBIASED analysis

**Fig. 1:**
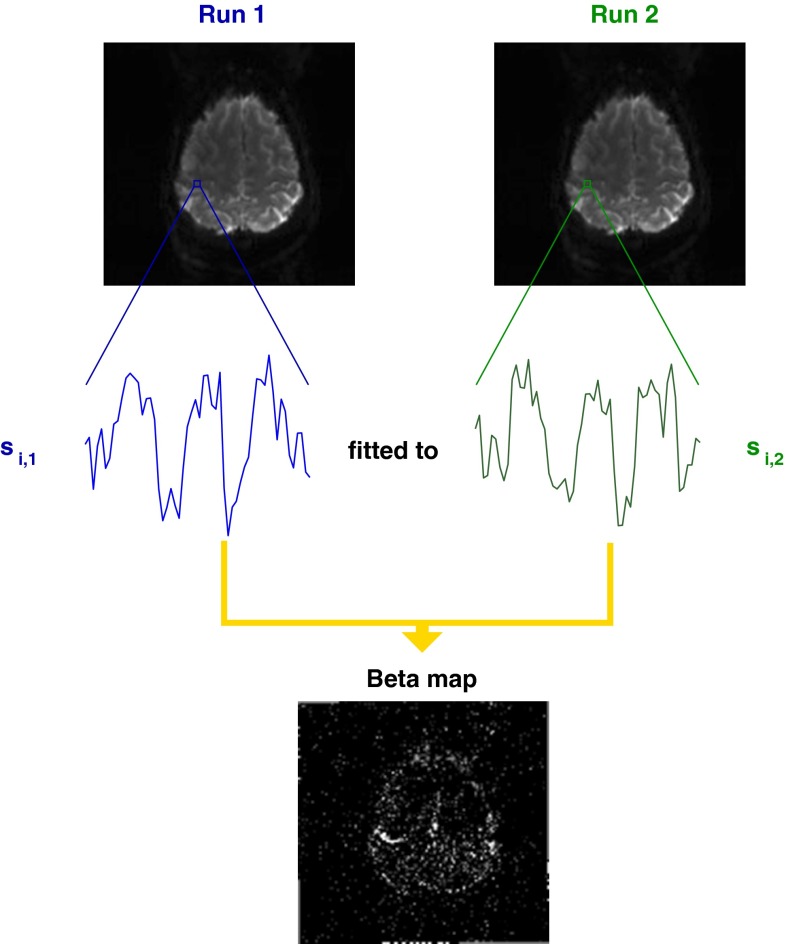
*Step 1* in the UNBIASED method illustrated for one pair of runs of a hand task. The time courses in the enlargements are from a single voxel, *i*, in a region activated by the task, which was presented in an ABABABA block design, where A was the rest phase and B a hand motor task. The beta value for each voxel is the result of the fit of the time series for that voxel in Run *j* to that in Run *k* (in this example, Run 1 to Run 2)

In contrast to the GLM, the “model” in UNBIASED is unique for every voxel: it is the time course in the same voxel in a different run. The first step in the analysis is illustrated for the simplest case, with two runs, in Fig. 1. A map of fit (beta) values is generated by fitting the time courses of each voxel in Run 1 to the time course of the corresponding voxels in Run 2. This is the same as Levin et al.’s BIASLESS method, other than that fit values are calculated (rather than cross–correlation values).

**Fig. 2:**
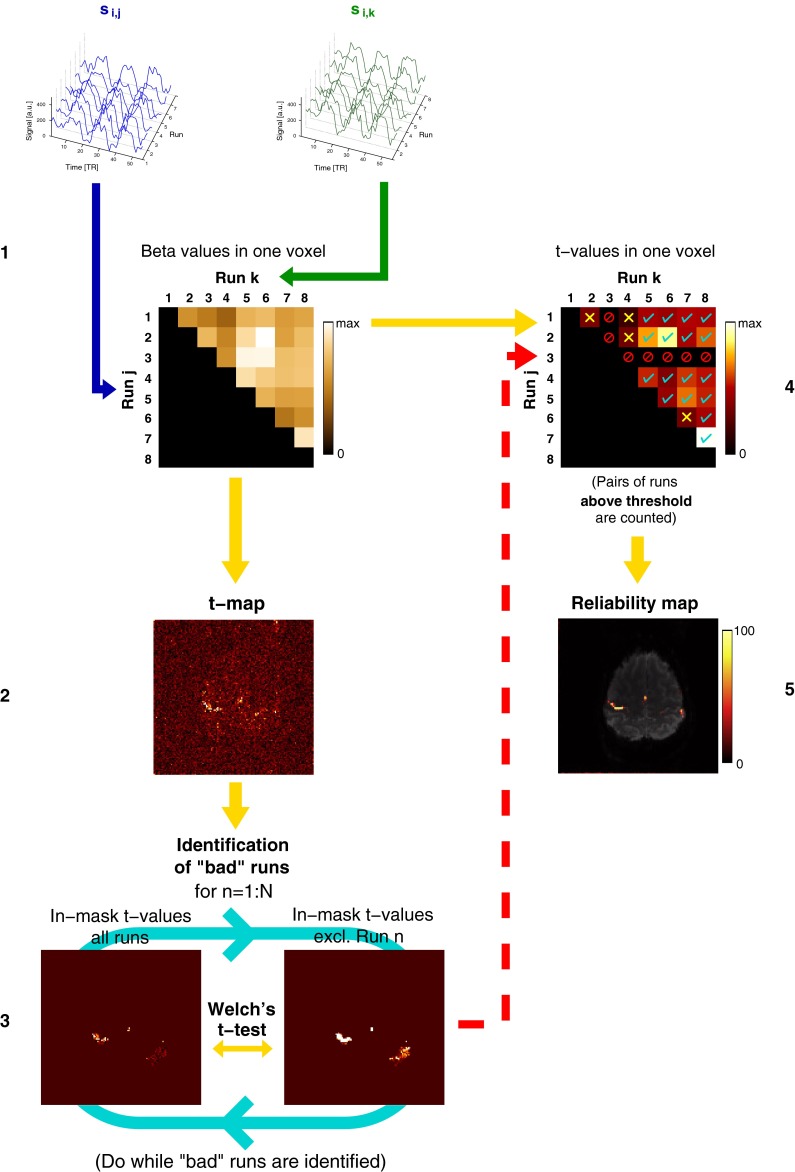
The main steps in UNBIASED, illustrated for eight runs of a hand task. Voxel-wise fit (beta) values are calculated between the time courses of all non-identical combinations of runs for each voxel (*Step* *1*). *Step* *2* For each voxel, *t* values are calculated from the beta values of all non-identical combinations of runs. *Step* *3* “Bad” runs are identified by performing a Welch’s *t* test between the t-map derived from all runs and that which excludes the run under consideration (*Run* *n*) (see “Identification of “bad” runs”). Run 3 is excluded in this example (red “forbidden” signs in *Step* *4*). Voxel-wise *t* values are thresholded at an uncorrected *p* < 0.001. Those *t* values exceeding this threshold are counted (cyan ticks in *Step* *4*). Those that fail to fulfil this criterion are marked with yellow crosses (*Step* *4*). From all the “good” pairs of runs, the proportion of supra-threshold *t* values to the total (in %) is used to generate the reliability map (*Step* *5*), the final result

The extension of this step to *N* runs, which leads to a matrix of beta values for each voxel, and further steps in UNBIASED, are illustrated in Fig. 2. They comprise:

(i) Voxel-wise calculation of beta and *t* values from fits between non-identical runs;
(ii) Identification of “bad” runs via a comparison of t-maps obtained with the inclusion and exclusion of each run; and
(iii) Assessment of how consistently each voxel is activated—the final activation measure.

These steps are explained in more detail in the following sections.

#### Voxel-wise beta and *t* values calculation

Pairwise linear least squares regression is calculated between the time courses of corresponding voxels in non-identical pairs of runs, via QR decomposition [32, 33]: the signal time course $s_{i,j} (t)$ of voxel $i$ and Run $j$, $j \in \left\{ {1, \ldots ,N - 1} \right\}$, is fitted to signal time course $s_{i,k} (t)$, $k \in \left\{ {j + 1, \ldots ,N} \right\}$. That is, the voxel time course of Run 1 is fitted to the corresponding voxel time course of Run 2, Run 3, etc., up to Run *N*, where *N* is the total number of runs. The procedure is repeated for Run 2, which is fitted to all runs from Run 3 to Run *N*, and so on for the other runs until Run *N*–*1*, which is fit to Run *N* only. Voxel-wise *t* values are calculated as the ratio of the fit (beta) value to the standard error for that particular voxel for each run combination considered, as defined by Woolrich et al. [34]. For each voxel, the beta and *t* values constitute triangular half matrices of non-identical run combinations (Fig. 2, Steps 1 and 4, respectively). A subject-level one sample *t* test is calculated from the beta values for all the non-identical combinations of runs using MATLAB’s “ttest” function at a *p* = 0.05 significance level (Fig. 2, Step 2). Results from this test are used in the Step “Identification of ‘bad’ runs”.

#### Identification of “bad” runs

Runs in which performance was poor or were affected by artefacts show lower *t* values in the main activation foci. In UNBIASED, these runs are identified by testing, for each Run *n*, the null hypothesis that Run *n* yields *t* values which do not differ from those calculated from other runs. For each *n*, the hypothesis is tested in a one-sided two-sample *t* test assuming unequal sample variances (Welch’s *t* test) in which the samples are taken from:

(i) The t-map generated from all the pairs of runs in Step 2 (Fig. 2), and
(ii) The t-map from all the pairs that exclude Run *n*, $n \in \left\{ {1, \ldots ,N} \right\}{\backslash }\left\{ n \right\}.$

The test is performed for voxels in an “activation” mask—a smoothed map of voxels for which the *t* values are in the top 1 percentile. A run is excluded if the null hypothesis is rejected at a significance level of 0.05, to which a Bonferroni correction for multiple comparisons is applied. If multiple runs are identified in one pass of this test, the one with the smallest *t* value is excluded. This process is repeated until no more runs are excluded (Fig. 2, Step 3).

#### Reliability map

The multiplicity of *t* values for each voxel (Fig. 2, Step 4) allows the generation of a map representing the reliability of a voxel to be activated. The reliability is calculated as the percentage of pairs of runs in which the *t* value for a given voxel exceeds an uncorrected *p* < 0.001 threshold to the total number of combinations of runs ($N \cdot \left( {N - 1} \right)/2$). This operation is performed for all voxels in runs classified as “good”. Run combinations that exceed the threshold are indicated with cyan ticks in Step 4 in Fig. 2. The final result in UNBIASED is a map of the percentage of pairs of good runs in which each voxel is activated, which we have called “Reliability map” (Fig. 2, Step 5).

### Participants

This study was approved by the ethics committee of the Medical University of Vienna. All participants provided written informed consent prior to inclusion.

Functional MRI data was acquired from six right-handed healthy subjects (mean age 28 ± 4 years old, 2 females) with no history of neurological, psychiatric, or psychological disorders. One of three purely motor paradigms (hand, chin, and foot movement) were performed by each of three volunteers (V1, V2, and V3, respectively) and a visuomotor paradigm by all of the remaining three (V4, V5, and V6).

### Task description

Volunteers V1, V2, and V3 were asked to perform 20 runs of one of three functional motor tasks (hand, chin, or foot movement). All the functional paradigms were performed in a block design. Each run consisted of four rest and three movement phases of 20 s each, presented in an ABABABA design (A: rest phase; B: task phase). Commands to commence and cease movement were communicated via visual cues—a circle which changed from red (rest) to green (task) at the centre of a white fixation crosshair on a black background.

The hand paradigm was a self-paced repetitive opening and closing of the dominant (right) hand with a frequency of approximately 1 Hz. The chin paradigm was a repetitive opening and closing of the mouth with a target of one open and close cycle per second. The foot paradigm consisted of alternated dorsal and plantar flexion of the right foot with a frequency of approximately 0.5 Hz.

Volunteers V4, V5, and V6 were instructed to perform a target of 30 runs of a visuomotor task in a block design modified from the paradigm used in Gonzalez-Castillo et al. [9] in the following manner: an initial rest period of 10 s was followed by five alternated repetitions of a task (10 s) and a rest (20 s) block. During the task, subjects were asked to concentrate on the centre of a flickering checkerboard (frequency = 7.5 Hz) and simultaneously open and close the dominant (right) hand with a frequency of approximately 1 Hz, and during rest to focus on a white crosshair on a black background and remain still. V5 completed only 20 runs. For subject V6, fMRI data was acquired for an additional run in which no task was presented (to enable the run exclusion feature to be tested).

Stimuli were presented using the software Presentation (Neurobehavorial Systems, Berkeley, CA, USA) and triggered by the MRI scanner.

### Data acquisition

Images were acquired with a 7 T Siemens MAGNETOM scanner (Siemens, Erlangen, Germany) and a 32-channel RF coil (Nova Medical, Wilmington, MA, USA).

Functional MRI data were acquired with a 2D single-shot gradient echo EPI sequence, with 30 slices of 3 mm thickness with a 0.3 mm gap acquired parallel to the Anterior Commissure–Posterior Commissure (AC–PC) plane, with a matrix size of 128 × 128, FOV = 220 × 220 mm (nominal 1.7 × 1.7 mm in-plane resolution), TE = 22 ms, TR = 2500 ms (V1, V2, and V3) and 1000 ms (V4, V5, and V6), and partial Fourier encoding of 3/4 (omission of the first 25 % of phase-encoding steps), receiver bandwidth of 1446 Hz/pixel, and parallel imaging with a GRAPPA factor of 2. For the visuomotor task, multiband acceleration 2 was also used. Three dummy scans were used in the runs with 56 volumes and seven dummy scans were used in the runs with 160 volumes to achieve quasi-equilibrium in longitudinal magnetization.

### Data preprocessing

**Fig. 3:**
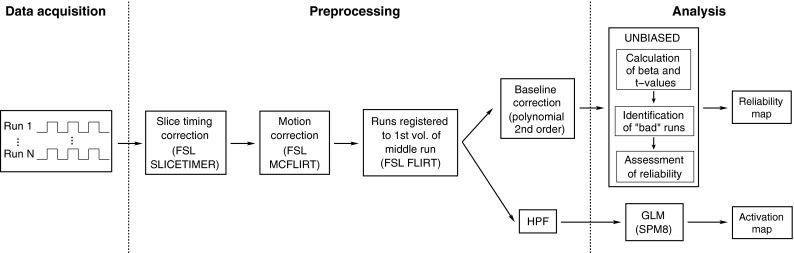
Schematic view of the steps applied to the fMRI data from acquisition to final activation/reliability maps

Acquisition, preprocessing and analysis steps are schematically illustrated in Fig. 3.

Analysis was performed on a single subject basis in the native space of the EPI, as is common practice in presurgical planning [29, 35–37]. EPI data was preprocessed with FSL [38], with the exception of a correction for baseline drift used in UNBIASED, which was performed in MATLAB. The fMRI time-series were slice timing corrected with SLICETIMER, except for the data from V4, V5, and V6, to which no timing correction was applied due to the short TR, and corrected for motion using MCFLIRT [39], with six degrees of freedom. Each run was registered to the first volume of the middle run using FLIRT [40], with 12 degrees of freedom. For UNBIASED, low frequency baseline signal drift was removed by subtracting a second-order polynomial fit to each voxel time series in each run (performed in MATLAB using a least mean squares method). For the GLM, default high pass filtering (HPF) was used instead. No normalization or spatial smoothing was performed.

### Assessment of UNBIASED features

#### Consistency of UNBIASED results

The consistency of UNBIASED results for three motor tasks (hand, chin, and foot movement) was assessed in the study of V1, V2, and V3, and compared with the consistency of the GLM. For each task, the split-half reliability was calculated: 20 acquired runs were split into two groups—the odd and the even runs—which were analysed separately with both UNBIASED and SPM. Similarity was assessed in the following manner:

(i) The number of voxels with a particular reliability value in UNBIASED in each group of runs, *N*_(Group)_{reliability} (e.g. *N*_odd_95), was counted.
(ii) For the GLM analyses of the same runs, the *N*_(Group)_{reliability} voxels with the largest *t* values were selected.
(iii) The extent of the activation overlap, or congruence, between the odd and the even runs for each task, reliability and analysis method was assessed using Dice’s coefficients [41]. Reliability values from 0 to 100 % were assessed in intervals of 5 %.

#### Effect of delayed response

In order to compare the sensitivity of UNBIASED and the GLM to consistent delays in response, temporal shifts of −7.5, −5.0, −2.5, 0.0, 2.5, 5.0, and 7.5 s (−3, −2, −1, 0, 1, 2, and 3 volumes) were artificially introduced post hoc into the voxel time series from the three motor tasks performed by V1, V2, and V3 prior to analysis with the two methods. Temporal shifts were attained by circularly shifting volumes in time series such that the final volumes were shifted to the beginning of the time series for positive shifts and the first volumes were shifted to the end of the time series for negative shifts.

#### Identification of “bad” runs

The effectiveness of the identification of “bad” runs in UNBIASED was assessed in all healthy subjects by examining the GLM results for each run. Where a “bad” run was identified in the subjects who performed the visuomotor task (V4, V5, and V6) a pseudo-randomised choice of nine runs in addition to the “bad” run were assessed. This is the typical target number of runs in presurgical planning. Additionally, subject-level results generated with and without exclusion of the runs identified as “bad” in UNBIASED were compared by calculating percentage changes in reliability values in a generous ROI (drawn by hand) containing activation in the primary motor area.

### GLM analysis

The subject data was analysed with the GLM (using SPM8) to provide a comparison with the UNBIASED results. Data were high-pass filtered (HPF) after preprocessing (as illustrated in Fig. 3) with a default cut-off frequency of 1/128 Hz. A canonical HRF was used, with no model derivatives or motion correction regressors. Only positive *t* values were considered.

#### Regions of modified response shape

UNBIASED is expected to provide improved detection of consistent non-model-conform responses. To allow these to be identified, the quality of fits between the model and the data were assessed for UNBIASED and an in-house implementation of the GLM (ihGLM) using data preprocessed identically (i.e. including baseline correction with a 2nd order polynomial). Voxel-wise goodness of fit was assessed using the coefficient of determination, $R^{2}$, using the general definition, $R^{2} \equiv 1 - {\text{SS}}_{\text{res}} / {\text{SS}}_{\text{tot}}$, where ${\text{SS}}_{\text{res}}$ and ${\text{SS}}_{\text{tot}}$ are the residual and total sum of squares from the fit, with ${\text{SS}}_{\text{res}} = \sum\nolimits_{i} {\left( {y_{i} - f_{i} } \right)^{2} }$ and ${\text{SS}}_{\text{tot}} = \sum\nolimits_{i} {\left( {y_{i} - \overline{y} } \right)^{2} }$, where $y_{i}$ and $f_{i}$, are the signal and predicted (or modeled) signal time courses and $\overline{y}$ the average signal over time per voxel, respectively. The ratio $r_{\text{UG}} = (R_{\text{UNBIASED}}^{2} - R_{\text{ihGLM}}^{2} )/(R_{\text{UNBIASED}}^{2} + R_{\text{ihGLM}}^{2} )$ was computed and clusters containing contiguous voxels with better goodness of fit in UNBIASED ($r_{\text{UG}} > 0$) were selected and investigated. Average time courses from cubic ROIs of size 5 × 5 × 5 voxels centred on these clusters were calculated and compared to the GLM regressor.

## Results

### UNBIASED features

#### Consistency of UNBIASED results

**Fig. 4:**
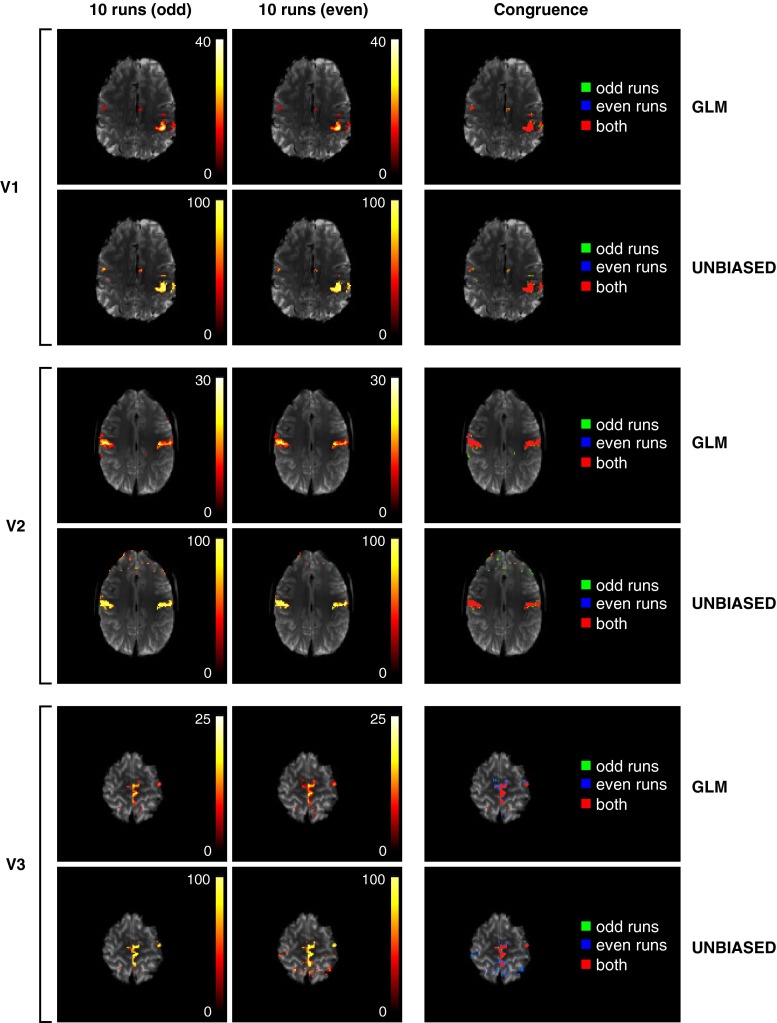
Consistency of GLM and UNBIASED results thresholded at 50 % reliability (see “Consistency of UNBIASED results”) for three motor paradigms (hand, chin and foot movement) for V1, V2, and V3. Images are displayed in radiological convention. *Left and centre columns* GLM and UNBIASED results for 10 odd and 10 even sets of runs. *Right column* Congruence in the voxels identified as activated in both the odd and even sets of runs (*red*), and activation identified only in odd runs (*green*) and even runs (*blue*)

GLM and UNBIASED results for the odd and even runs of the hand, chin, and foot motor tasks, thresholded at 50 % reliability (see “Consistency of UNBIASED results”) are shown in Fig. 4 (left and centre columns, respectively), and demonstrate a high level of consistency between the two groups of runs. Congruence maps for the three tasks indicate the extent of overlap in results at the chosen threshold (Fig. 4, right). There was congruence (red voxels) between results from the odd and even runs in the primary motor cortex (M1) and the supplementary motor area (SMA) in all tasks. Congruence in the SMA is only shown in Fig. 4 for the foot task, but was also present in higher slices for the hand and chin tasks. At lower thresholds, some congruent false positive voxels were visible due to stimulus-correlated motion, particularly in the frontal lobe in the chin task and at high contrast boundaries such as the gyri and sulci in the parietal lobe in the foot task (not shown). The similarity of the results from odd and even runs was assessed with Dice’s coefficients, the value of which were, for the GLM {V1: 0.88, V2: 0.69, V3: 0.59} and for UNBIASED {V1: 0.87, V2: 0.66, V3: 0.54} at a threshold of 50 % reliability. The average difference between Dice’s coefficients for the GLM and UNBIASED calculated over all assessed thresholds was 0.07 for all tasks, indicating similar congruence in GLM and UNBIASED results.

#### Effect of delayed response

**Fig. 5:**
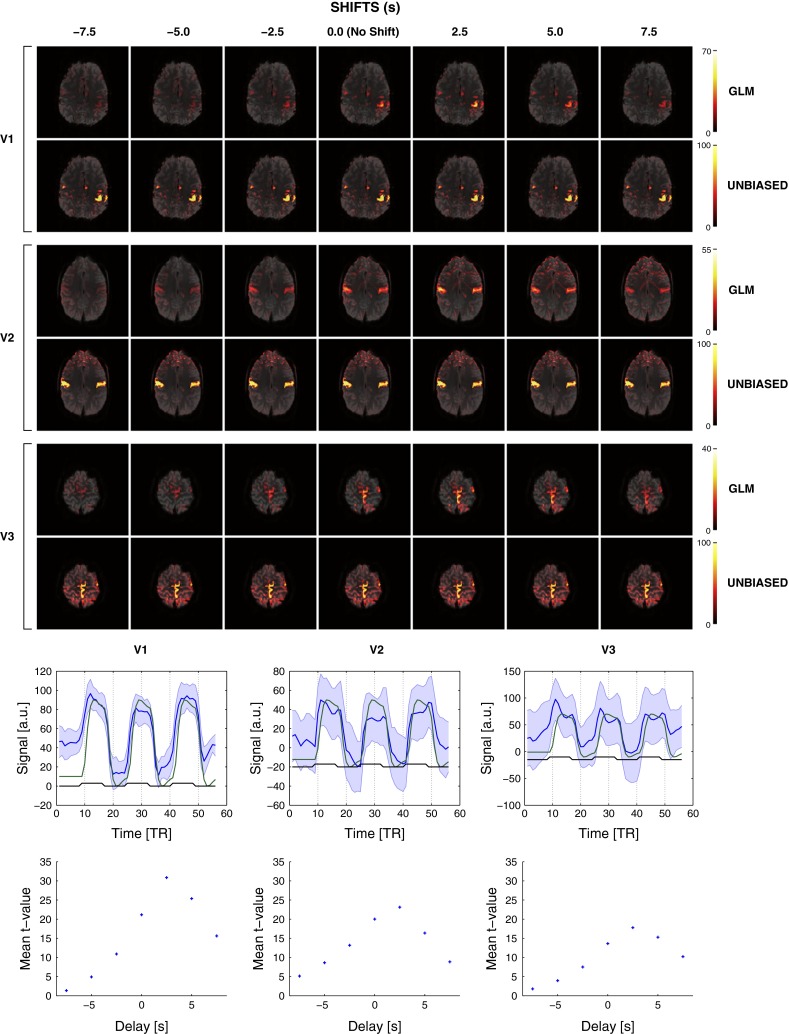
*Top* Effect of delayed responses in GLM (*top rows*) and UNBIASED (*bottom rows*) analyses illustrated for the hand, chin, and foot tasks. The impact on magnitude and extent of activation for shifts of 2.5 s (1 TR) in the time courses from −7.5 to 7.5 s are shown. Overlays are presented with a transparency of 25 % in radiological convention. *Bottom* (*top row*) Plots of the average time courses (*blue line*) and standard deviation (*blue shaded area*) in a cubic ROI centred at the activation area (no time course shift) for all tasks. *Green and black lines* represent the GLM regressor and task timing, respectively. *Bottom row* Average *t* values calculated in the same ROI for time course shifts of 2.5 s from −7.5 to 7.5 s. For all tasks, the highest average *t* values were obtained for a shift of +2.5 s

The sensitivity of GLM and UNBIASED to response delays was assessed for three motor tasks, to which time delays (with respect to the measured data) of −7.5, −5.0, −2.5, 0.0, 2.5, 5.0, and 7.5 s (−3, −2, −1, 0, 1, 2, and 3 volumes) were added. The resulting reliability maps (UNBIASED) and t-maps (GLM) for all motor tasks are shown in Fig. 5 (top). Average GLM *t* values in M1 for the hand task (V1) were (listing shift then corresponding *t* value): {(−7.5 s: 1.4), (−5.0 s: 4.9), (−2.5 s: 10.9), (0.0 s: 21.2), (2.5 s: 30.8), (5.0 s: 25.4), (7.5 s: 15.6)}. That is, negative shifts led to reduced *t* values. The highest *t* value was for a positive shift of +2.5 s in all tasks, indicating that the response in the original (unshifted) data occurred earlier (by circa 2.5 s) than specified in the model (Fig. 5, bottom). UNBIASED results, in contrast, showed the expected consistency in the reliability of activation, irrespective of shifts in task timing. Findings were similar for the chin and foot tasks (V2 and V3, respectively).

#### Identification of “bad” runs

**Fig. 6:**
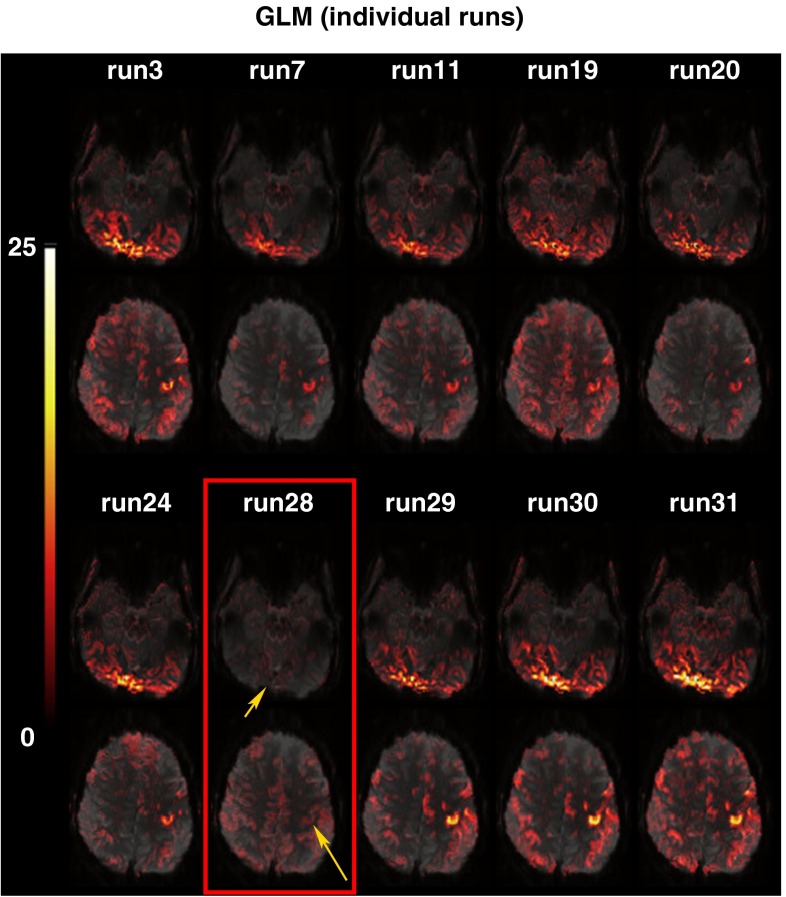
Assessment of a run identified by UNBIASED as being of low quality in volunteer V6 (Run 28). The pairs of overlays for each run show a slice in visual (*top*) and motor (*bottom*) areas. GLM results for each run (positive *t* values) show weak/absent activation in Run 28 (*yellow arrows*). Images are presented with a transparency of 25 % in radiological convention

UNBIASED identified one compromised run in one volunteer (V6) that corresponded to the run in which no stimulus was presented and, therefore, no task was performed. From the 31 runs measured in this volunteer, 10 (including the compromised run) were chosen pseudo-randomly for demonstration purposes. GLM analysis of the individual runs (Fig. 6) shows little activation (at yellow arrows) in the run identified as “bad” by UNBIASED (Run 28), and better results in other runs. Exclusion of this run from the UNBIASED calculation with the 10 chosen runs resulted in reliability values that were 21 % higher in the activated area.

### Comparison of GLM and UNBIASED

**Fig. 7:**
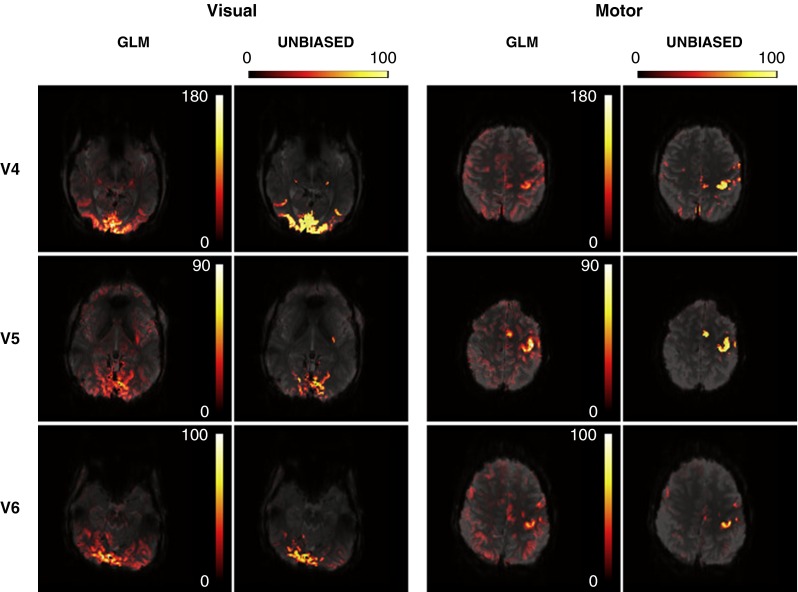
A comparison of visuomotor localizations in three volunteers (V4, V5, and V6) using GLM t-maps (positive *t* values) (*1st and 3rd columns*) and unthresholded UNBIASED reliability maps (*2nd and 4th columns*). Visual activation (*columns 1 and 2*) and motor activation (*columns 3 and 4*) was detected with both methods for these volunteers. UNBIASED results generally suffered less from artefact contamination. Overlays are presented with a transparency of 25 % in radiological convention

Positive GLM *t* values and unthresholded UNBIASED results for V4, V5, and V6 are shown in Fig. 7. Results for V1, V2, and V3 can be seen in Fig. 5 (column “No Shift”). It was possible to identify the motor cortex in both GLM and UNBIASED results in all volunteers and the visual area in V4, V5, and V6 (subjects performing a visuomotor task). UNBIASED results suffered from less artefact contamination than the GLM in most cases (V1, V4, V5, and V6). However, both GLM and UNBIASED results showed higher artefact contamination in frontal and parietal areas for V2 and V3, respectively. The tasks that these subjects performed (chin and foot movement) elicited the most head motion.

#### Regions of modified response shape

**Fig. 8:**
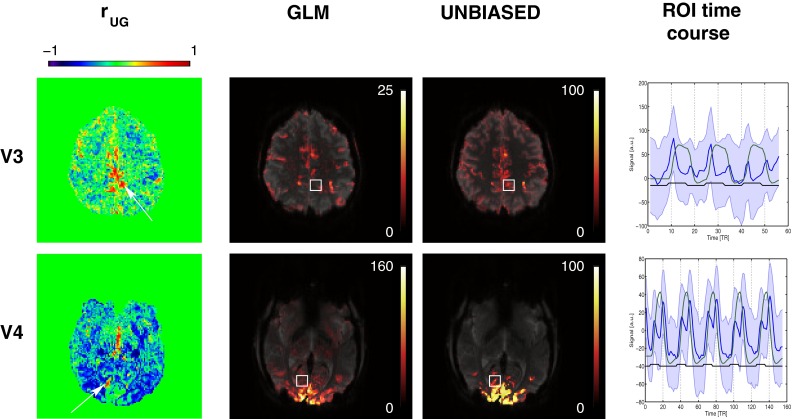
*Left column* Maps of $r_{\text{UG}}$ for two volunteers who had regions with modified response shapes and discrepancies between GLM and UNBIASED results. *White arrows* point to clusters of $r_{\text{UG}} > 0$ (better quality of fit in UNBIASED). *Middle columns* GLM (*left*) and UNBIASED (*right*) activation and reliability maps in radiological convention (25 % transparency). *Right column* Plots of the mean (*blue line*) and standard deviation (*blue shaded area*) time courses in cubic ROIs (*white boxes* centered at the clusters with $r_{\text{UG}} > 0$). *Green lines* represent the GLM model regressor and *black lines* the stimulus timing

Regions with reproducibly modified response shape were identified in two volunteers (V3 and V4). Aside from the sustained smooth response present in a large extent of the visual and motor cortices, temporal signal changes time-locked with the task were observed in these areas, as illustrated in Fig. 8. Average time courses extracted from cubic ROIs centered in these regions reveal transient signal changes that occurred primarily during task-switching periods (i.e. at the start and/or end of the task block). These non-model-conform responses led to a lower fit quality and thus reduced BOLD sensitivity in the GLM. The sensitivity of UNBIASED was uncompromised in those regions, with reliability values in the approximate range 50–80 %.

## Discussion

The fMRI analysis method described here, UNBIASED, is an extension of the BIASLESS approach of Levin and Uftring [23], which allows the integration of information from a number of runs.

UNBIASED is based only on the assumption that the signal time course in activated voxels does not vary significantly between repetitions of the same experiment, making it capable of detecting non-model-conform BOLD responses. In this study, which was focused on motor tasks presented in a manner commonly used in presurgical planning, UNBIASED was shown to yield highly reproducible activation maps with low artefact contamination, accurately identify runs of low quality and detect BOLD signal changes in regions in which the response deviated significantly from model predictions. Results from preliminary data acquired in two healthy volunteers performing an overt speech task in a block design with the same timing as the motor tasks shows that the method is also effective in cognitive functional regions (e.g. Broca and Wernicke areas responsible for speech processing), which produce smaller and less reliable signal changes than motor or visual areas (Supporting Fig. 1).

Increasing the fMRI duration (e.g. via the number of runs) increases the statistical power of the analysis and enables the reliability of activation to be assessed.

The acquisition of multiple runs introduces the possibility to automatically identify low quality runs and provide a measure of how reliably each voxel is activated by the task. In addition, it allows patients short rest periods between runs, which facilitates compliance and reduces the likelihood that patients move during the acquisition period. In fMRI for presurgical planning, the number of runs to be acquired is generally determined by patient compliance (usually assessed by a clinician) and how challenging the detection of activation is in the affected cortical area. UNBIASED was shown to be able to accurately depict activation with a number of runs as low as two (the minimum required for inter-run fitting of voxel time courses). The gradation (or number of intervals) of reliability values increases with the number of runs (see “Reliability map”), however. For instance, there are only two reliability levels (0 and 100) if two runs are used. If the number of runs is low, average beta (fit) values could be used to aid the user in distinguishing true activation from artefacts (Supporting Fig. 2).

The consistency of UNBIASED was assessed by determining the congruence between results from two sets of runs of each task, evaluating the extent of overlap in activation via Dice’s coefficients. The main limitation of cluster overlap methods is that they are dependent on the chosen statistical threshold that defines what is “active”. Here, we assessed results over a range of thresholds down to the lowest reliability value in UNBIASED and compared these with the corresponding number of most highly activated voxels in the GLM. UNBIASED was found to have a similar consistency to that of the GLM over all thresholds.

Unlike the GLM, UNBIASED does not make assumptions about the temporal dynamics of BOLD signal changes. As such, it was expected to perform well in cases involving modified response shape, consistently compromised task performance or modified HRF. Interestingly, in our analysis of the sensitivity of GLM and UNBIASED to response delays, the highest BOLD sensitivity in the GLM did not occur for no delay but for a delay of 1 volume (+2.5 s). Closer examination of the time course of activation revealed a consistent negative delay in time-to-onset and time-to-peak of the response of roughly 1 TR (2.5 s) with respect to the GLM regressor in all the tasks investigated, making it clear that BOLD sensitivity in the GLM can be suboptimal even with healthy subjects and simple sensorimotor stimuli. This is consistent with prior studies of healthy volunteers reporting delays in latency of 3–14 s [42–45]. A shift of just −1 volume (−2.5 s), on the other hand, led to substantially lower GLM *t* values, even with the block design used in this study, which has been adopted for presurgical planning due to its robustness to time shifts. The GLM would be expected to be still more sensitive to response delays in experiment designs with shorter blocks or events.

The quality of fMRI data may be compromised by technical artefacts, poor performance or motion. Some of these factors can be reduced by training, but task execution needs to be closely monitored and failures in paradigm execution documented. Data quality—the absence of artefacts due to motion, drift, slice timing error, ghosting, etc.—should also be assessed prior to analysis [46]. As well as being onerous, this process runs the risk of overlooking artefacts that present atypically and of excluding data, which is useful despite some contamination. UNBIASED offers a fully automated means of identifying runs, which do not contribute to the quality of the subject-level analysis either because of technical artefacts, motion, or poor performance. The capability of UNBIASED to automatically identify and exclude “bad” runs from the analysis via comparative Welch’s *t* tests is particularly desirable in clinical fMRI, as the results need to be available shortly after the examination in order to insure that they can be considered in the therapeutic decision-making process.

Modified response shape and magnitude have been reported by Gonzalez-Castillo et al. [9], where BOLD signal modulation consistent with task timing was found to extend beyond areas of primary relationship to the task. In our study, UNBIASED was able to detect such regions (whose timing was time-locked to the task but with consistently modified response shape) which were not present in GLM results. In brain tumours, a decrease in amplitude [4, 5] and a delay in onset of the HRF have been reported [14]. Similar findings were observed in cerebrovascular diseases due to changes in vascular reactivity [15]. Brain arteriovenous malformations (AVMs) represent a typical condition in which regional cerebral blood flow (rCBF) and perfusion is significantly reduced [16], modifying the HRF. As was shown in this study, UNBIASED is insensitive to consistent response shape and timing effects, suggesting that robust results may be achieved despite atypical temporal hemodynamics. UNBIASED is expected to be equally sensitive to activation in areas which arise due to pathology-associated reorganization. The response in newly formed functional areas is presumed to be consistent between runs, even if it differs in shape from that in other areas.

ICA has been applied to presurgical planning data to identify non-model-conform activation foci and separate activation from artefacts [22]. In ICA, spatio-temporal relationships in the data are split into components that are conventionally ordered by the percentage of total explained signal variance in the data they explain. In the presence of motion, fluctuating Nyquist ghosts, and accelerated acquisition artefacts, task-related activation is often a minor contributor to total variance in the data, meaning that a large number of components need to be assessed by the user. Methods to rank the components on the basis of spatial and temporal features (e.g. correlation between frequency spectra of model time courses and frequency spectra of ICs) have been suggested to ease identification of task-related activation components [22]. However, temporal features have shown poor performance due to the similarity between the frequency spectra of activation and stimulus-correlated motion. Spatial features, on the other hand, require additional analysis and/or co-registration of the functional data to anatomical or functional templates, which can be challenging, particularly in clinical cases. Furthermore, task related ICs may still be split in two or more components, requiring further user intervention. UNBIASED generates a single activation map representing all reproducible signal changes regardless of shape or sign, unifying, in many contexts, the model-free advantages of ICA with the simplicity of interpretation of the GLM.

A measure based on reliability is of crucial importance in a clinical context, where localization uncertainties are critical. Using a model-based analysis, Beisteiner et al. [26] showed that a combination of a voxel reliability measure (from multiple runs) with a high correlation threshold contribute to a reduction in false positive and false negative activation. Likewise, UNBIASED makes use of the information in multiple runs to derive a measure of reliability. This may provide indication about the degree of involvement a particular area possesses in task execution and aid the surgeon in the decision about resection margins or necessity of alternative treatment strategies.

The main limitations of UNBIASED are that it requires the fMRI experiment to be structured as multiple runs with the same timing and that, in the current implementation, it is not capable of separating activation from multiple tasks. Theoretically, physiological fluctuations could lead to high reliability values between scans if the phases and frequencies of the oscillations were sufficiently similar between runs and the runs were to start at the same phase of the physiological cycles. In practice, however, the start of runs is unrelated to physiological fluctuations and these are themselves subject to such variation over the scanning period that no coherent relationship exists between runs. Hence, physiological noise and motion uncorrelated with the task are artefact sources to which UNBIASED is highly insensitive, which is desirable.

UNBIASED may be more prone to motion artefacts than the GLM if task-evoked motion is present (as observed in the chin and foot tasks). Such artefacts are sometimes observed in high-contrast tissue boundaries such as brain/air and grey/white matter interfaces. In patients, these could additionally arise close to tumour or oedema borders. In UNBIASED, these would be expected to be present even if event-related designs are used, whereas stimulus-correlated motion and the BOLD response can be distinguished in a GLM analysis due to the temporal separation between the two [47]. Finally, UNBIASED has been shown to identify activation where time courses are consistent between runs. This advantage is not maintained if there is variation in response dynamics between runs, due to changes in the timing of task execution or the use of different mental strategies during several acquisitions, because of learning or adaptation.

## Conclusion

Functional MRI at ultra-high field strength benefits from increased time-series SNR, BOLD sensitivity [29], and specificity to BOLD signal changes in the microvasculature [48, 49]. These advantages may serve clinical populations by improving the reliability with which activation is detected [28–30] or allowing the measurement time to be reduced. As well as applying the most suitable measurement methods and correction strategies to secure the advantage of UHF fMRI, this study demonstrates that applying robust analysis methods improves the prospect of realising the full clinical potential of UHF fMRI.

The UNBIASED fMRI analysis method generates activation maps that are highly consistent over multiple executions of the same task. In this study, UNBIASED activation maps were generally less contaminated by false positive activation than those from a GLM analysis. UNBIASED results agreed well with GLM t-maps for both motor and visuomotor tasks. The challenge of controlling for technical artefacts, motion, and poor task performance in fMRI can be mitigated by the possibility of automatically identifying and excluding low quality runs.

The ability to identify consistent, but atypical BOLD responses is a valuable feature when compromised task performance or modified HRF is expected. This makes it particularly interesting as a complementary approach for neurologists and neurosurgeons in presurgical planning, to determine the necessity and extent of resection of pathological brain tissue.

## Electronic supplementary material

Below is the link to the electronic supplementary material.

Supplementary material 1 (PDF 397 kb)
