# Supplementary material for: Improving the clinical potential of ultra-high field fMRI using a model-free analysis method based on response consistency
Source: MAGMA. 2016 Mar 10;29:435–49. doi: 10.1007/s10334-016-0533-8 (PMC4891377; doi:10.1007/s10334-016-0533-8)
Supplement: Supplementary file 1 — Supplementary material 1 (PDF 397 kb) [file 10334_2016_533_MOESM1_ESM.pdf]

SUPPORTING MATERIAL

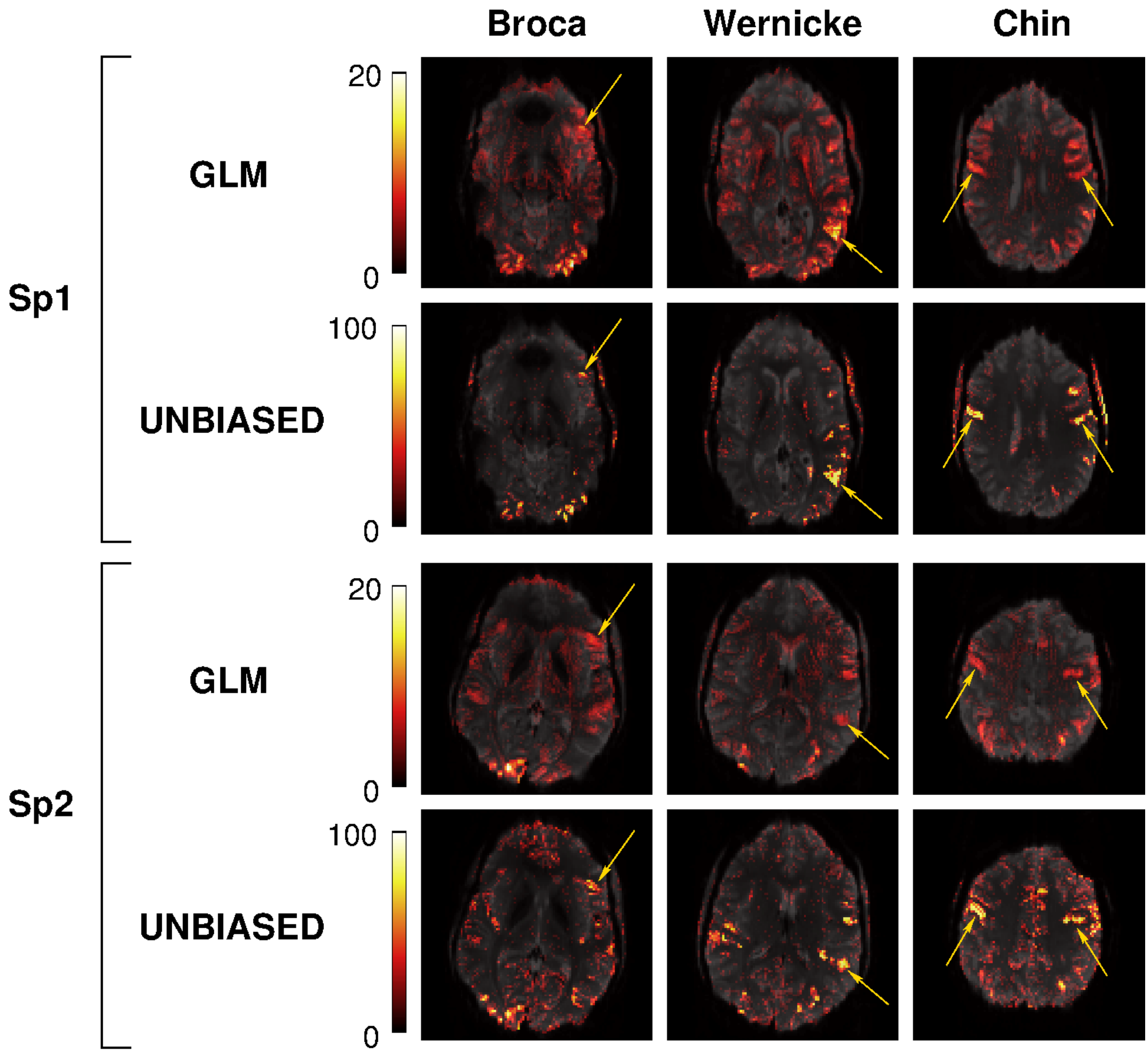

Supporting Fig. 1 GLM (*1st and 3rd rows*) and UNBIASED (*2nd and 4th rows*) results for two healthy volunteers performing an overt speech task in an ABABABA block design with the same timing as the motor tasks in this study. Slices containing functional areas responsible for speech processing (*1st and 2nd columns*) and chin motion (*3rd column*) are shown. Results indicate that the method is also effective in cognitive functional regions, which produce smaller and less reliable signal changes than motor or visual areas (e.g. Broca and Wernicke areas responsible for speech processing).

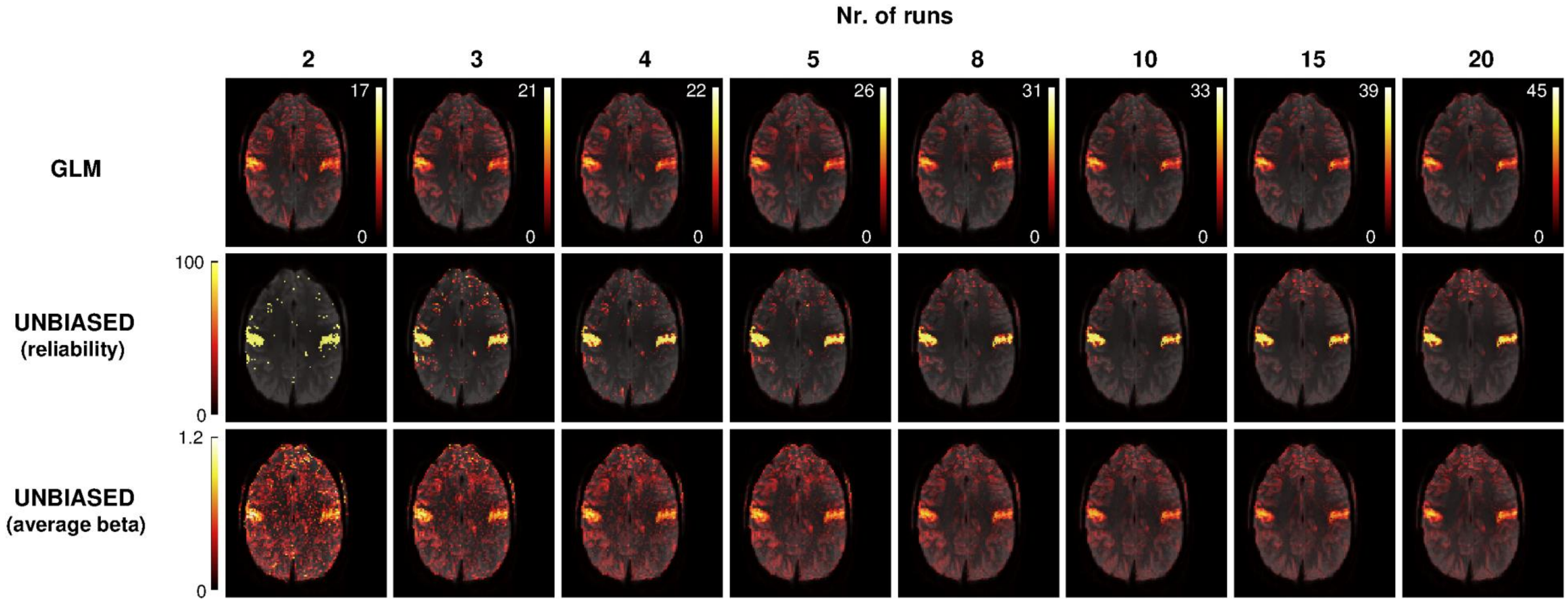

Supporting Fig. 2 Effect of increasing the number of runs in the GLM (*top row*) and UNBIASED (*middle row*) analyses of data from a healthy volunteer performing a chin task. *Bottom row*: UNBIASED average beta (fit) values. Results indicate that UNBIASED is able to accurately depict activation with a number of runs as low as two (the minimum required for inter-run fitting of voxel time courses). The gradation (or number of intervals) of reliability values increases with the number of runs (e.g. there are only two reliability levels (0 and 100) if two runs are used). If the number of runs is low, average beta (fit) values could be used to aid the user in distinguishing true activation from artefacts.
